# Relationship between the duration of smoking and blood pressure in Han and ethnic minority populations: a cross-sectional study in China

**DOI:** 10.1186/s12889-020-09975-w

**Published:** 2021-01-14

**Authors:** Yuelun Zhang, Yunying Feng, Shi Chen, Siyu Liang, Shirui Wang, Ke Xu, Dongping Ning, Xianxian Yuan, Huijuan Zhu, Hui Pan, Guangliang Shan

**Affiliations:** 1 Medical Research Center, Peking Union Medical College Hospital, Chinese Academy of Medical Sciences and Peking Union Medical College, Beijing, China; 2 Peking Union Medical College Hospital, Chinese Academy of Medical Sciences and Peking Union Medical College, Beijing, China; 3 Department of Endocrinology, Peking Union Medical College Hospital, Chinese Academy of Medical Sciences and Peking Union Medical College, Beijing, China; 4 Department of Endocrinology, The First Affiliated Hospital of Wenzhou Medical University, Wenzhou, China; 5 Department of Pediatrics, Linfen Central Hospital, Linfen, China; 6 Department of Epidemiology and Statistics, Institute of Basic Medical Sciences, Chinese Academy of Medical Sciences, School of Basic Medicine, Peking Union Medical College, Beijing, China

**Keywords:** Blood pressure, Smoking, Chinese populations, Minority groups, Cross-sectional study

## Abstract

**Background:**

Evidence for correlation between the cigarette use and blood pressure change remains ambiguous. This study modelled relationship between the duration of smoking and systolic blood pressure in a large national multi-ethnic cross-sectional survey in China.

**Methods:**

Participants were selected through a multi-stage probability sampling procedure from 2012 to 2017. Former or current smokers were included in this study, whose smoking behaviour, blood pressure, and other demographic information were collected and measured through a face-to-face interview. Linear and non-linear relationships between the duration of smoking and systolic blood pressure were analysed and differences of the association between Han and minority populations were specially checked.

**Results:**

A total of 8801 participants were enrolled in this study. Prevalence of hypertension was 41.3 and 77.8% were current smokers. For every additional year of smoking duration, systolic blood pressure raised by 0.325 mmHg (95% CI 0.296 to 0.354 mmHg, *P* <  0.001). The Chinese minority populations may suffer more from the elevated blood pressure in long-term smoking than Han populations (0.283 mmHg (95% CI 0.252 to 0.314 mmHg, *P* <  0.001) versus 0.450 mmHg (95% CI 0.380 to 0.520 mmHg, *P* <  0.001) raise in systolic blood pressure with each additional year of smoking in minority and Han populations).

**Conclusions:**

Smoking is associated with raised systolic blood pressure in Chinese population. This association is notedly stronger in Chinese minority populations.

## Background

Hypertension, also known as high blood pressure, is a major contributor to many serious non-communicable diseases, such as coronary heart disease and chronic kidney disease [1]. In 2016, approximately 34% of men and 6% of women beyond 15 years old all over the world were current tobacco smokers [2]. The association between cigarette smoking and blood pressure is still unclear. Cigarette smoking was found to be associated with the increased risk of hypertension [3–5] with a dose-response relationship [6–9], while this association was not found in some population-based studies [10].

Ethnic or racial variations in abnormal blood pressure have been noticed [11, 12]. These ethical variations are in particular important in China due to a diversity of ethnic groups (the Han and other 55 ethnic minority populations) and socioeconomic conditions in different regions. A large national cross-sectional survey in China reveals that hypertension prevalence, awareness, treatment, and control have obvious dissimilarities in different ethnic populations [13]. In addition to these variations in the hypertension, the minority ethnic populations also have a significantly higher prevalence of smoking [14]. Despite of these differences, previous studies focusing on the effect of smoking on blood pressure did not evaluate if the association between smoking and blood pressure differs in the Han and other ethnic minority populations.

Given the controversial associations found in previous studies, this study aimed to analyse the relationship between the duration of smoking and systolic blood pressure in Han and ethnic minority smoking populations based on a large national cross-sectional survey conducted in China. The potential non-linear relationship was specially checked and the association patterns in Han and ethnic minority smoking populations were further compared.

## Methods

### Ethics, study registration, and reporting

The study was approved by the Bioethical Committee of the Institute of Basic Medical Sciences, the Chinese Academy of Medical Sciences, Beijing, China (No. 028–2013). Written informed consent was obtained from all the participants. There is no online registration or published research protocol for this study. The study is reported in accordance with the STROBE statement [15].

### Study design and population sampling

The study was conducted based on the China National Health Survey (CNHS), a large population-based cross-sectional survey that involved in 11 provinces in China. The overall design and rationale of the survey have been reported previously [16, 17]. A total of 53,895 participants aged from 20 to 80 years were recruited through a multistage, stratified cluster sampling procedure. CNHS provides a representative sample of the Chinese population, in particular reflecting multi-ethnicity. The field survey was initiated in October 2012 in Guizhou Province, while the last eligible population was recruited from Hebei Province in 2017. For this study, we applied for and obtained 37,567 records from Yunnan Province, Gansu Province, Guizhou Province, Hainan Province, Heilongjiang Province, Inner Mongolia Autonomous Region, Shaanxi Province, and Xinjiang Autonomous Region.

### Inclusion and exclusion criteria

Eligible participants of this study were those who had been recorded as current or ever smokers in the CNHS. Participants with incomplete data on the starting or cessation age of smoking, measured blood pressure, sex, alcohol use, body mass index (BMI), or physical activity were excluded.

### Data collection and measurements

Trained staff from the local Center for Disease Control and Prevention collected the information during face-to-face interviews. Demographic information, including age, sex, ethnic group, physical activity, and health-related behaviours (tobacco and alcohol use), was collected by a standard questionnaire (Web Table 1, see Additional file 1), which was developed for this study. Current smokers were defined as smoking at least 1 cigarette per day in the past 6 months. Former smokers were defined as having quit smoking for more than 6 months at the time of the survey. Detailed consumption of cigarette or tobacco leaf was also collected. Alcohol drinking was defined as consuming at least 30 g of alcohol per day for at least 6 months. Former alcohol drinking was defined as having stopped drinking for more than 6 months at the time of the survey. Physical activity was classified into light, moderate, and heavy groups according to the participants’ daily labour work. Diabetes was diagnosed using the criteria proposed by the World Health Organization in 1999 [18], and hypertension was diagnosed if the systolic blood pressure was equal to or larger than 140 mmHg, or the diastolic blood pressure was equal to or larger than 90 mmHg.

A physical examination was conducted to collect the body data of each participant. Participants were asked to rest for 5 min before blood pressure measurements. Blood pressure was measured 3 times with 1-min intervals. The mean value of these 3 measurements was set as the final blood pressure value. Standing height and weight were also measured. Body mass index (BMI) was calculated by the weight (unit: kilogram) divided by the square of the height (unit: metre). An 8-h overnight fasting blood sample was collected to measure the fasting glucose and lipid levels.

### Definition of the exposure, confounding, and outcome variables

The main exposure in this analysis was the duration of smoking, calculated by the current age minus the starting age of smoking. If the participant had stopped smoking, the duration of smoking was defined as the stopping age minus the starting age. This study primarily analysed the systolic blood pressure since it is a stronger predictor for cardiovascular events compared to other blood pressure measures.

Except duration of smoking, factors that may have a causal relationship with systolic blood pressure were regarded as potential confounders and adjusted in the multiple regression models. Based on previous studies and clinical experiences, sex, alcohol use, BMI, physical activity, low-density lipoprotein cholesterol (LDL-C), and fasting plasma glucose (FPG) were adjusted. Age was not adjusted in the multivariable model due to the strong correlation between the actual age and duration of smoking (Pearson’s correlation = 0.767, *P* <  0.001), which would lead to severe problem of collinearity in the modelling.

### Statistical analysis

Basic characteristics of participants were tabulated and differences between Han and ethnic minority populations were compared using t-test and chi-square test. Association between the duration of smoking and systolic blood pressure was firstly checked by linear regression model. We built 2 adjusted models together with the crude model to evaluate the association between the duration of smoking and systolic blood pressure.

**Table:** 

Model 1:	*Systolic blood pressure*	~	*Duration of smoking*
Model 2:	*Systolic blood pressure*	~	*Duration of smoking + sex + alcohol drinking + BMI + physical activity*
Model 3:	*Systolic blood pressure*	~	*Duration of smoking + sex + alcohol drinking + BMI + physical activity + LDL-C + FPG*

Interpretation of the findings was mainly based on the fully adjusted model 3. The residuals were plotted against the predicted values to check the goodness of fit of the linear models. Points uniformly and randomly distributed around the horizontal line at 0 were considered as a suitable fit to the observations.

Restricted cubic spline model was used to check if there existed non-linear relationship between the duration of smoking and systolic blood pressure. In the spline function, 3 knots were set, whose locations were estimated based on the quantile of the duration of smoking. The coefficient of the non-linear section in the spline model were specifically checked and a two-sided *P* value less than 0.05 was considered as a statistically significant coefficient of the non-linear relationship. Segmented model was further used if the duration of smoking and systolic blood pressure showed non-linear relationship, in order to find the potential breaking point of the association. To avoid overfitting, we allowed only 1 breaking point in the segmented model if there was no complex non-linear relationship judged by the scatter plot. Point estimate of the breaking point, together with the corresponding standard error, was yielded by the model through analysing the change of the linear relationship [19].

Predefined subgroup analysis was primarily conducted by the ethnic groups. Participants were divided into Han population and ethnic minority populations. Models mentioned above were repeated in Han and ethnic minority populations, and results were compared between the populations. We employed the method proposed by D. Altman to check whether the differences of breaking points yielded by segmented models were statistically significant [20].

We conducted 5 sensitivity analyses to check the robustness of our findings. First, the potential confounding factors were included in the adjusted spline as well as segmented models to check if the non-linear relationship still existed. Second, age-stratified analysis by the quantiles was performed to control for the potential confounding effect from age. Third, we modelled the cumulative tobacco exposure, instead of the duration of smoking, in the fully adjusted linear model. The cumulative tobacco exposure was calculated by the number of cigarette consumption per year multiplied by the duration of smoking. If the person used hand-made wrapped tobacco leaf instead of the cigarette, each 1 g leaf consumption was regarded as 1 cigarette use in the analysis. Fourth, diastolic blood pressure, instead of systolic blood pressure, was modelled to check if the association still existed. Last, due to the low prevalence of smoking in female population, the female participants were excluded to check if the association still existed in the male population.

Statistical analysis was completed in R (version 4.0.2., R Foundation for Statistical Computing, Vienna, Austria, 2020, https://www.R-project.org/) with “segmented” [21], “spline2” [22], “ggplot2” [23], and “rms” packages [24]. A two-sided *P* value less than 0.05 was regarded as statistically significant.

## Results

**Fig. 1:**
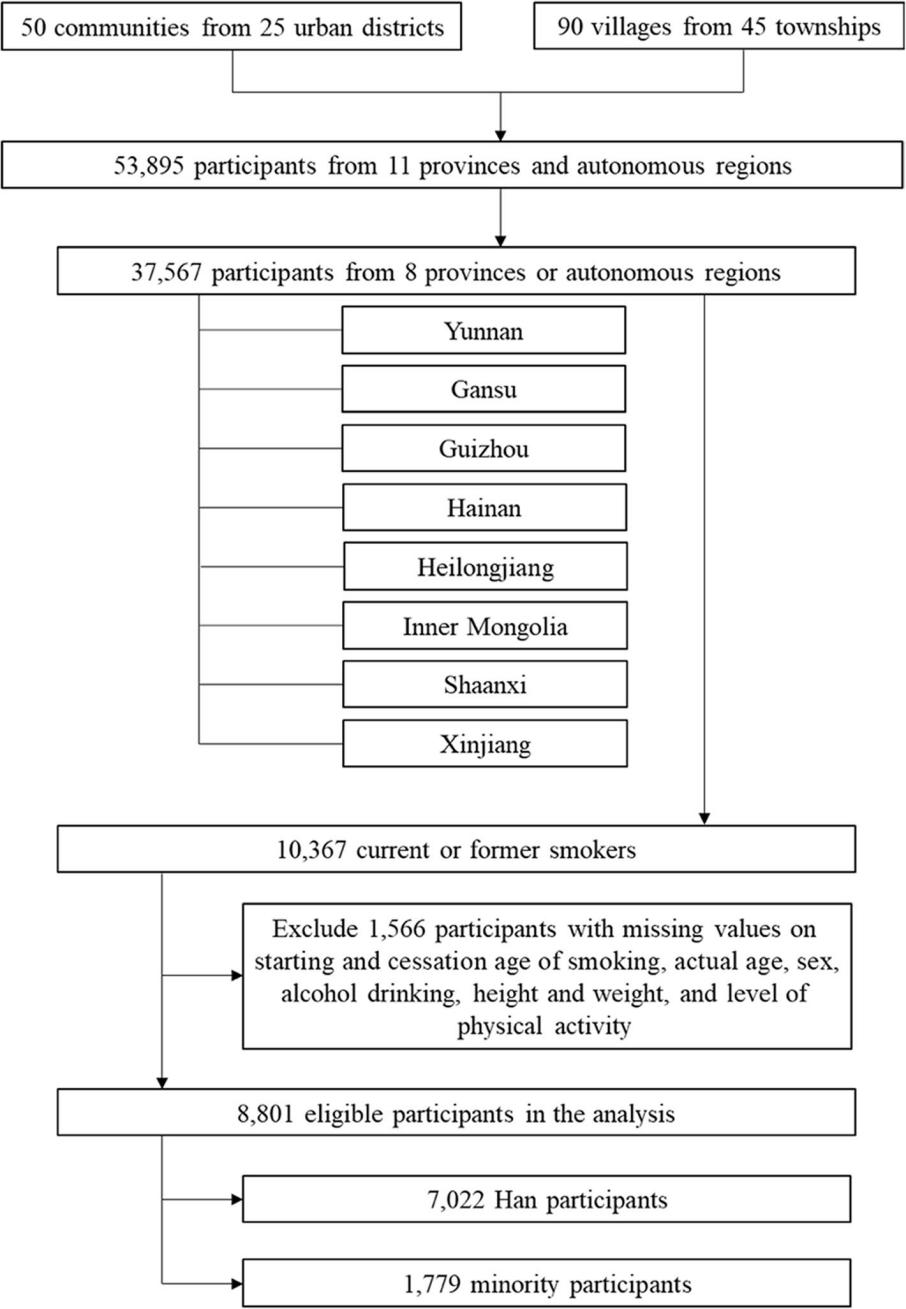
Flow chart of the participants inclusion and exclusion

The CNHS collected 37,567 participants from Yunnan Province, Gansu Province, Guizhou Province, Hainan Province, Heilongjiang Province, Inner Mongolia Autonomous Region, Shaanxi Province, and Xinjiang Autonomous Region, 10,367 of whom were current or ever smokers. After excluding the participants with missing values on starting and cessation age of smoking, actual age, sex, alcohol drinking, height, weight, or level of physical activity, a total of 8801 records were included in our analysis. The detailed participant inclusion and exclusion are shown in Fig. 1.

Basic characteristics of eligible participants are shown in Table 1. Mean age was 49.55 years old. And most eligible participants were male, which is in line with the fact that male smokers are more common than female smokers in China. Mean BMI was 23.79 and 41.3% of participants were diagnosed with hypertension or had elevated blood pressure according to the blood pressure measurements in the field survey. 77.8% were current smokers while 22.2% had quitted smoking. The mean duration of smoking was 25.92 years. Four ethnic groups, including Han (7022, 79.8%), Uyghur (656, 7.5%), Yi (215, 2.4%), and Bouyei (908, 10.3%), were recruited in the analysis. Compared with Han population, ethnic minority populations had fewer female smokers and worse health-related risk factors, such as higher BMI, higher blood pressure and glucose, more current smokers, longer duration of smoking, and more alcohol drinkers. More ethnic minority participants were involved in heavy physical labour work.

**Table 1:** Characteristics of the participants

Variable	Total	Han population	Minority population	***P***
Sample size	8801	7022	1779	–
Age (year)	49.55 (13.63)	49.63 (13.53)	49.21 (14.04)	0.252
Sex				
Male	8431 (95.8%)	6681 (95.1%)	1750 (98.4%)	< 0.001
Female	370 (4.2%)	341 (4.9%)	29 (1.6%)	
BMI	23.79 (3.82)	23.72 (3.73)	24.04 (4.13)	0.004
LDL-C (mmol/L)	2.82 (0.87)	2.82 (0.88)	2.85 (0.87)	0.206
FPG (mmol/L)	5.38 (1.49)	5.42 (1.51)	5.22 (1.38)	< 0.001
Diagnosed with hypertension based on medical record or measured SBP/DBP				
Yes	3633 (41.3%)	2683 (38.2%)	950 (53.4%)	< 0.001
No	5168 (58.7%)	4339 (61.8%)	829 (46.6%)	
Diagnosed with type 2 diabetes based on medical record or measured fasting glucose				
Yes	1810 (20.6%)	1213 (17.3%)	597 (33.6%)	< 0.001
No	6991 (79.4%)	5809 (82.7%)	1182 (66.4%)	
Smoke				
Quit	1956 (22.2%)	1631 (23.2%)	325 (18.3%)	< 0.001
Current	6845 (77.8%)	5391 (76.8%)	1454 (81.7%)	
Duration of smoking (year)	25.92 (12.91)	25.84 (12.80)	26.23 (13.37)	0.270
Drink				
Never	2239 (25.4%)	1847 (26.3%)	392 (22.0%)	< 0.001
Quit	1014 (11.5%)	743 (10.6%)	271 (15.2%)	
Current	5548 (63.0%)	4432 (63.1%)	1116 (62.7%)	
Physical activities				
Light	4843 (55.0%)	4197 (59.8%)	646 (36.3%)	< 0.001
Moderate	1552 (17.6%)	1268 (18.1%)	284 (16.0%)	
Heavy	2406 (27.3%)	1557 (22.2%)	849 (47.7%)	
Systolic blood pressure	127.92 (18.28)	127.22 (17.48)	130.66 (20.90)	< 0.001
Diastolic blood pressure	78.65 (11.64)	78.75 (11.41)	78.22 (12.50)	0.104

The participants were described using means with standard deviations and numbers with percentage. *P* values were calculated based on the comparisons between the Han population and the minority population

*Abbreviations*: *CI* Confidence interval, *BMI* Body mass index, *LDL-C* Low-density lipoprotein cholesterol, *FPG* Fasting plasma glucose

**Fig. 2:**
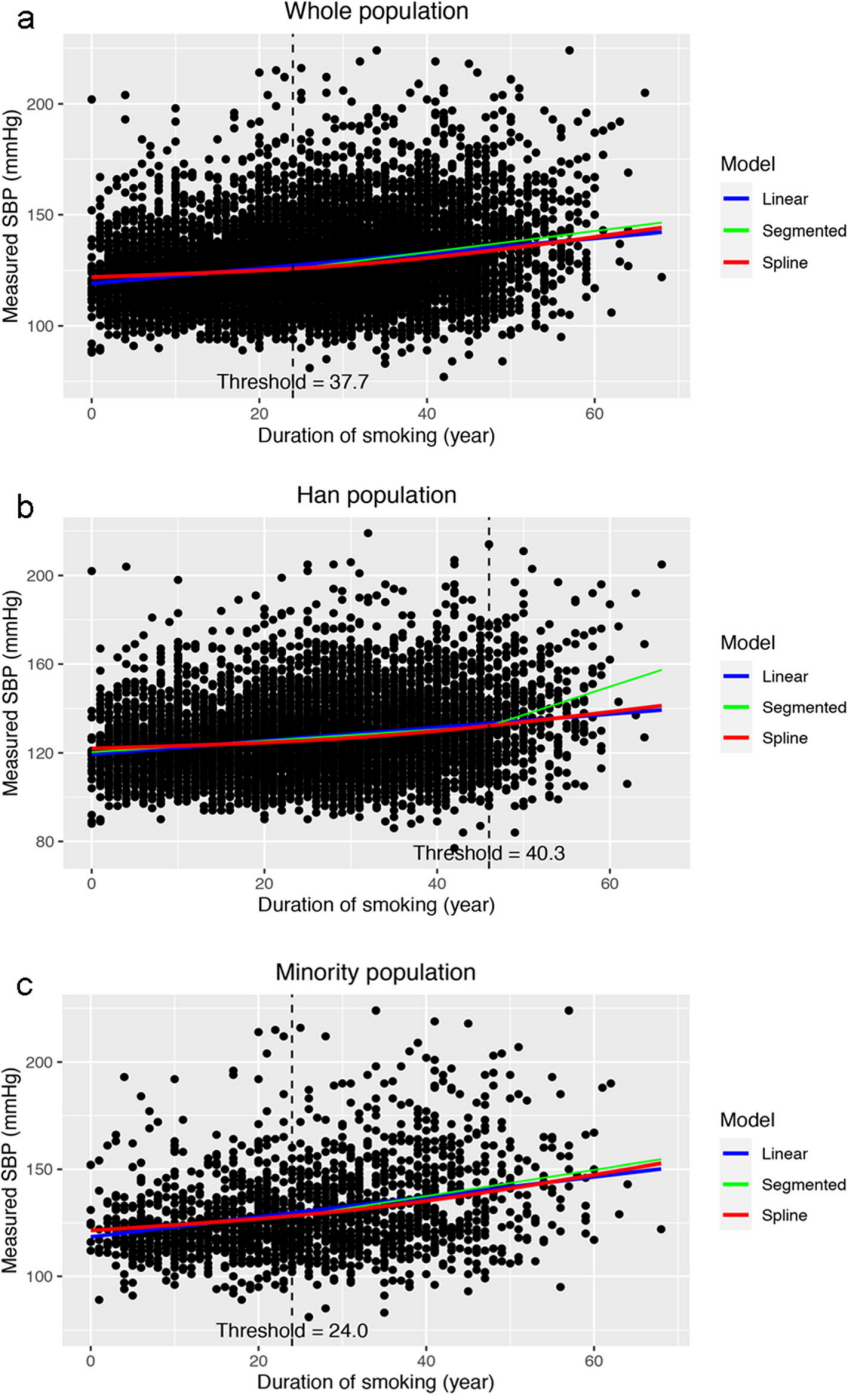
Models for the association between duration of smoking and systolic blood pressure. Fitted linear, spline, and segmented models by R. **a** The whole population. **b** The Han population. **c** The minority populations. SBP, systolic blood pressure

Duration of smoking was found to be associated with systolic blood pressure in the whole population by linear regression model. After adjusting for sex, alcohol drinking, BMI, physical activity, LDL-C, and FPG, for every additional year of smoking duration, systolic blood pressure raised by 0.325 mmHg (95% CI 0.296 to 0.354 mmHg, *P* <  0.001, see Table 2). This association was also found in other models and the regression coefficients were quite similar (crude model: 0.339, 95% CI 0.311 to 0.368, *P* <  0.001; model adjusting for confounders except for LDL-C and FPG: 0.352, 95% CI 0.323 to 0.380, *P* <  0.001), indicating a robust association. Residual plot in Web Figure 1 (see Additional file 1) did not provide evidence for severe violation of the modelling assumptions. A potential non-linear relationship between the duration of smoking and systolic blood pressure is shown in Fig. 2a). The scatter plot and regression line indicate that smoking may have a greater impact on systolic blood pressure in people with longer smoking duration (blue line).

**Table 2:** Models for the duration of smoking and systolic blood pressure in the whole population

Model	Estimate	95% CI	***P***
**Linear model**			
Model 1			
Duration of smoking	0.339	0.311, 0.368	< 0.001
Model 2			
Duration of smoking	0.352	0.323, 0.380	< 0.001
Sex (male vs. female)	1.700	−0.160, 3.560	0.073
Quit vs. never drink	1.147	−0.162, 2.456	0.086
Current vs. never drink	3.084	2.209, 3.959	< 0.001
BMI	0.701	0.605, 0.797	< 0.001
Moderate vs. light physical activity	−3.164	−4.171, − 2.157	< 0.001
Heavy vs. light physical activity	−0.742	−1.602, 0.119	0.091
Model 3			
Duration of smoking	0.325	0.296, 0.354	< 0.001
Sex (male vs. female)	2.117	0.265, 3.968	0.025
Quit vs. never drink	0.695	−0.608, 1.998	0.296
Current vs. never drink	3.011	2.141, 3.881	< 0.001
BMI	0.637	0.541, 0.733	< 0.001
Moderate vs. light physical activity	−2.929	−3.931, −1.928	< 0.001
Heavy vs. light physical activity	−0.240	−1.099, 0.620	0.585
LDL-C	1.188	0.768, 1.608	< 0.001
FPG	1.165	0.916, 1.414	< 0.001
**Spline model**			
Model 4 ^a^			
Duration of smoking (linear)	0.136	0.066, 0.205	< 0.001
Duration of smoking (non-linear)	0.254	0.175, 0.333	< 0.001
**Segmented model**			
Model 5^a^			
Threshold of duration of smoking	37.694	34.185, 41.202	–
Duration of smoking (shorter than 37.694 years)	0.261	0.218, 0.304	< 0.001
Duration of smoking (longer than 37.694 years)	0.696	0.543, 0.849	–

*Abbreviations*: *CI* Confidence interval, *BMI* Body mass index, *LDL-C* Low-density lipoprotein cholesterol, *FPG* Fasting plasma glucose

^a^ Crude model

Restricted cubic spline model tested the hypothesis that there existed the non-linear relationship. The statistical significance of non-linear section in the spline model was achieved as *P* value was less than 0.001. Plotting the prediction values of systolic blood pressure in the spline model against the duration of smoking indicates that smoking duration may contribute a stronger effect on systolic blood pressure in participants with longer duration of smoking (Fig. 2a), red line). Segmented model yielded a breaking point at 37.69 years (95% CI 34.19 to 41.20, Table 1, Model 5) and the association of duration of smoking and systolic blood pressure was significantly stronger in participants with more than 37.69-year history of smoking (Fig. 2a), green line).

The association between the duration of smoking and systolic blood pressure was also found in Han and ethnic minority populations separately. For every additional year in the duration of smoking, systolic blood pressure raised by 0.283 mmHg (95% CI 0.252 to 0.314 mmHg, *P* <  0.001) in Han population and 0.450 mmHg (95% CI 0.380 to 0.520 mmHg, *P* <  0.001) in ethnic minority populations (Table 3 Model 3 and Table 4 Model 3). The correlation of ethnic minority populations was statistically significantly stronger than that of Han population (*P* for interaction between duration of smoking and ethnicity < 0.001). Non-linear association was found in both Han and ethnic minority populations. In Han population, the association was statistically stronger after 46.00 years (95% CI 43.59 to 48.42 years, Table 3 Model 5 and Fig. 2b) green line) of smoking, while this breaking time was much earlier in the ethnic minority populations (23.95 years, 95% CI 14.71 to 33.18 years, Table 4 Model 5 and Fig. 2c) green line).

**Table 3:** Models for the duration of smoking and systolic blood pressure in the Han population

Model	Estimate	95% CI	***P***
**Linear model**			
Model 1			
Duration of smoking	0.302	0.271, 0.334	< 0.001
Model 2			
Duration of smoking	0.318	0.287, 0.349	< 0.001
Sex (male vs. female)	2.548	0.685, 4.411	0.007
Quit vs. never drink	1.787	0.351, 3.223	0.015
Current vs. never drink	2.498	1.570, 3.427	< 0.001
BMI	0.831	0.725, 0.936	< 0.001
Moderate vs. light physical activity	−2.707	−3.762, −1.651	< 0.001
Heavy vs. light physical activity	−2.570	−3.548, − 1.593	< 0.001
Model 3			
Duration of smoking	0.283	0.252, 0.314	< 0.001
Sex (male vs. female)	3.195	1.343, 5.047	0.001
Quit vs. never drink	1.276	−0.151, 2.704	0.080
Current vs. never drink	2.359	1.438, 3.280	< 0.001
BMI	0.752	0.646, 0.857	< 0.001
Moderate vs. light physical activity	−2.476	−3.523, -1.429	< 0.001
Heavy vs. light physical activity	−2.144	−3.115, −1.172	< 0.001
LDL-C	1.597	1.149, 2.045	< 0.001
FPG	1.184	0.921, 1.446	< 0.001
**Spline model**			
Model 4 ^a^			
Duration of smoking (linear)	0.118	0.043, 0.193	0.002
Duration of smoking (non-linear)	0.234	0.147, 0.320	< 0.001
**Segmented model**			
Model 5^a^			
Threshold of duration of smoking	46.006	43.594, 48.418	–
Duration of smoking (shorter than 46.006 years)	0.256	0.220, 0.292	< 0.001
Duration of smoking (longer than 46.006 years)	1.262	0.869, 1.655	–

*Abbreviations*: *CI* Confidence interval, *BMI* Body mass index, *LDL-C* Low-density lipoprotein cholesterol, *FPG* Fasting plasma glucose

^a^ Crude model

**Table 4:** Models for the duration of smoking and systolic blood pressure in the minority population

Model	Estimate	95% CI	***P***
**Linear model**			
Model 1			
Duration of smoking	0.468	0.398, 0.537	< 0.001
Model 2			
Duration of smoking	0.461	0.391, 0.531	< 0.001
Sex (male vs. female)	−8.390	−15.646, −1.134	0.023
Quit vs. never drink	− 1.153	−4.221, 1.915	0.461
Current vs. never drink	4.199	1.919, 6.480	< 0.001
BMI	0.225	0.001, 0.449	0.049
Moderate vs. light physical activity	−5.709	−8.487, −2.931	< 0.001
Heavy vs. light physical activity	0.583	−1.455, 2.620	0.575
Model 3			
Duration of smoking	0.450	0.380, 0.520	< 0.001
Sex (male vs. female)	−8.576	−15.811, − 1.341	0.020
Quit vs. never drink	−1.219	−4.283, 1.845	0.435
Current vs. never drink	4.282	2.007, 6.558	< 0.001
BMI	0.198	−0.027, 0.423	0.085
Moderate vs. light physical activity	−5.303	−8.079, −2.527	< 0.001
Heavy vs. light physical activity	1.092	−0.970, 3.154	0.299
LDL-C	0.155	−0.922, 1.233	0.778
FPG	1.302	0.633, 1.972	< 0.001
**Spline model**			
Model 4 ^a^			
Duration of smoking (linear)	0.259	0.081, 0.437	0.004
Duration of smoking (non-linear)	0.269	0.057, 0.481	0.013
**Segmented model**			
Model 5^a^			
Threshold of duration of smoking	23.948	14.714, 33.183	–
Duration of smoking (shorter than 23.948 years)	0.252	0.036, 0.469	0.022
Duration of smoking (longer than 23.948 years)	0.610	0.467, 0.752	–

*Abbreviations*: *CI* Confidence interval, *BMI* Body mass index, *LDL-C* Low-density lipoprotein cholesterol, *FPG* Fasting plasma glucose

^a^ Crude model

In sensitivity analysis, the non-linear correlation was still statistically significant after controlling for potential confounding factors in the adjusted spline models (*P* <  0.001). Detailed results from the adjusted spline and segmented models are shown in Web Table 2 (see Additional file 1). The fully adjusted linear regression model (Model 3) was conducted in the subgroups by the quantiles of the actual age of participants. The relationship between the duration of smoking and systolic blood pressure was found in the participants under 40 years old (every additional year of smoking was associated with 0.095 mmHg increase, 95% CI 0.016 to 0.174, *P* = 0.018, in systolic blood pressure) and over 60 years old (every additional year of smoking was associated with 0.101 mmHg increase in systolic blood pressure, 95% CI 0.024 to 0.178, *P* = 0.010), but not in other subgroups. Detailed results by the quantiles of age are shown in Web Table 3 (see Additional file 1). Analysis of the cumulative tobacco exposure shows that with each 50 packs increase of cigarette use per year (nearly 1 more pack per week) was associated with 0.014 mmHg (95% CI 0.012 to 0.017 mmHg, *P* <  0.001) raise of systolic blood pressure (Web Table 4, see Additional file 1). Crude and adjusted linear and non-linear models revealed that the duration of smoking was also significantly associated with the diastolic blood pressure (Web Table 5, see Additional file 1). Excluding female participants from the main analysis did not change the association significantly. Duration of smoking was still significantly associated with increased systolic blood pressure (every additional year of smoking was associated with 0.322 mmHg increase, 95% CI 0.292 to 0.351, *P* <  0.001).

## Discussion

Our analysis reveals that the duration of smoking, reflecting the cumulative tobacco exposure, was found to be non-linearly associated with elevated systolic blood pressure in the Chinese smoking population. This association was stronger in Chinese minority populations. The adverse effect of smoking was stronger after 37.7 years of smoking, while this breaking point was significantly earlier in Chinese minority populations than Han populations. All these findings consistently indicate that the Chinese minority populations are more likely to suffer from elevated blood pressure in the long-term smoking.

Despite the established relationship between cardiovascular outcomes and cigarette smoking [25], previous researches reveal controversial evidence for the effect of smoking cessation on blood pressure [26]. A large meta-analysis combining 23 population-based studies in Europe and the United States using Mendelian randomization to exclude the potential confounding effect reveals that current smoking was associated with lower blood pressure compared with non-smokers [27]. This protective effect was found in Asian populations [28], nevertheless, the adverse effect of smoking on raised blood pressure was meanwhile reported in China [29]. These inconsistent findings were explained by the effect of cigarette smoking on decreased body weight in some studies, because cigarette smoking was found to be causally associated with lower BMI [30], which led to the following decrease of blood pressure [31, 32].

The potential confounding effect from body weight cannot totally explain the inconsistent findings, because most of the previous studies have adjusted BMI or weight in their primary analyses. Our findings clearly support the existence of the association between smoking and raised systolic blood pressure. This association can be explained by the increased sympathetic nerve activity due to the nicotine and fine particulate matter in tobacco smoking [33] and the process of atherosclerosis [34]. The vasoconstriction, renin-angiotensin-system activation, and sodium and water absorption promoted by elevated sympathetic activity and cytokines would ultimately cause severe hypertension [35]. Also, the bidirectional influence of nicotine on inflammatory pathways through different nicotinic acetylcholine receptors could change the immune balance, leading to excessive inflammation and development of hypertension [36, 37]. We believe one important and significant difference between our analysis and previous studies is that duration of smoking, rather than the categories of smoking status, was modelled. Simply dividing the smoking status into non-smokers, former smokers, and current smokers, which was commonly used in previous analyses, ignored the detailed cumulative effect of smoking. Although requiring stronger assumptions and more complex models in the analysis, modelling the dosage of smoking exposure as a continuous variable can reflect the detailed association at each level of exposure and avoid loss of variance coming with the transformation into a categorial variable. Significant heterogeneity may exist in the same categorical group of smoking, for example, a current smoker with only 1-year history of cigarette smoking has more similar cumulative effects with a non-smoker rather than a current smoker with 20-year history of smoking. The potential health effects of smoking, if exists, should be associated with the cumulative exposure of the smoking. We suggest that further studies on smoking should consider using exposure level rather than smoking status in the data analysis.

Few previous studies used the cumulative exposure of smoking in the analysis, hence, the non-linear relationship between smoking and blood pressure was rarely reported. The non-linear relationship found in our analysis indicates that the adverse effect of smoking on blood pressure is stronger after long-term exposure of cigarette smoking. The interventions targeting the smoking cessation should specially focus on those with long history of cigarette smoking because this population may suffer more from elevated blood pressure. This does not mean that people with relatively short-term exposure of smoking are safe, because each 1-year increase in the duration of smoking is still statistically significantly associated with 0.26 mmHg raise (95% CI 0.22 to 0.30) in systolic blood pressure.

We observed significantly stronger association between smoking and raised systolic blood pressure in Chinese minority populations than Han populations. There were some discussions about the differences of prevalence, biological characteristics, and the following cardiovascular risks among different ethnical populations in western countries [38, 39]. Nevertheless, similar studies on Chinese minority ethnical populations are still quite limited. A cross-sectional survey conducted in Inner Mongolia (one of the autonomous regions where the Mongolian population is the main minority ethnicity) in China found that the current smoking status was only associated with hypertension in the Mongolian population, not in the Han population [40]. Although this study found a protective effect of smoking on hypertension which disagreed our findings, the heterogeneous association in different ethnicity populations was similar with our results. Numerous biological and pathological factors, such as various genetic background, may contribute to the stronger association found in minority ethnical populations, but we believe not only the biological factors but also the social-economic factors are potential reasons for the different associations.

This large national population-based study evaluated the linear and non-linear association between the duration of smoking and the systolic blood pressure. Ethnical differences of the association were specially checked. Main findings were based on statistical models adjusting for major confounders. However, this study may still have some limitations. Since our analysis was based on a cross-sectional survey, we cannot exclude the possibility of the reverse causality. It is unclear whether the elevated systolic blood pressure happened after the cigarette smoking, or the participants stopped smoking after they were diagnosed with hypertension. Future prospective studies can help to solve this problem. We analysed each 1 g wrapped tobacco leaf as 1 cigarette in the sensitivity analysis. However, as the crude tobacco leaf may vary a lot in different regions, it is possible that this transformation cannot reflect the exact dose of tobacco exposure. Previous studies have reported racial and ethnic disparities in responses to blood pressure lowering pharmacotherapy [41, 42], while pharmacological evidence on Chinese minority populations remains insufficient. This cross-sectional study could not accurately assess the effect of anti-hypertensive treatment, which would be of great significance in blood pressure control. We failed to include the concurrent anti-hypertensive treatments in the analysis, which may serve as an important confounder. If the heavy cigarette use was associated with worse compliance of anti-hypertensive treatments while the anti-hypertensive treatments can strongly lower the blood pressure, the association between smoking and blood pressure can be overestimated. The potential confounding effect from actual age of participants was not excluded in the sensitivity analysis. It is still possible that the aging process itself, rather than the tobacco, mainly contribute to the observed effect of the cumulative tobacco exposure. Multivariable models in the age quantile subgroups in the sensitivity analysis may still suffer from the residual confounding effect. Although the association between duration of smoking and systolic blood pressure was statistically significant, the effect size was relatively small and may be clinical irrelevant, which limits the external validation of our results. It is also difficult to totally decouple the effect of the cumulative tobacco exposure from the effect of aging itself, which severely limited the confidence of inference.

Further studies on cumulative exposure effects of smoking are required to clarify unsolved problems. On the population average level, the Han population had higher income levels and were more economically advantaged with better access to the healthcare services. Further healthcare decisions should consider the disparities and enforce the lifestyle interventions in the Chinese minority populations. We also recommend smoking cessation for all current smokers given the findings from our analysis and other potential health benefits.

## Conclusions

This study found that cumulative tobacco exposure is associated with raised systolic blood pressure in Chinese population, especially in the minority population.

## Supplementary Information

**Additional file 1: Web Figure 1.** Residual plot for checking the assumptions in the linear model in the whole population. **Web Table 1.** Questionnaire used in the field survey. **Web Table 2.** Adjusted non-linear models for the relationship between the duration of smoking and systolic blood pressure in the whole population. **Web Table 3.** Linear regression models for the relationship between the duration of smoking and systolic blood pressure in subgroups by the quantiles of age. **Web Table 4.** Adjusted linear model for the relationship between the cumulative tobacco exposure and systolic blood pressure in the whole population^a^. **Web Table 5.** Linear and non-linear models for the relationship between the duration of smoking and diastolic blood pressure in the whole population.

## Data Availability

The datasets used and analysed during the current study are available from the corresponding author on reasonable request.

## Abbreviations

BMI: Body mass index
CI: Confidence interval
CNHS: China National Health Survey
DBP: Diastolic blood pressure
FPG: Fasting plasma glucose
IQR: Interquartile range
LDL-C: Low-density lipoprotein cholesterol
SBP: Systolic blood pressure
