# Supplementary material for: Relationship between the duration of smoking and blood pressure in Han and ethnic minority populations: a cross-sectional study in China
Source: BMC Public Health. 2021 Jan 14;21:135. doi: 10.1186/s12889-020-09975-w (PMC7807505; doi:10.1186/s12889-020-09975-w)
Supplement: Supplementary file 1 — Additional file 1: Web Figure 1. Residual plot for checking the assumptions in the linear model in the whole population. Web Table 1. Questionnaire used in the field survey. Web Table 2. Adjusted non-linear models for the relationship between the duration of smoking and systolic blood pressure in the whole population. Web Table 3. Linear regression models for the relationship between the duration of smoking and systolic blood pressure in subgroups by the quantiles of age. Web Table 4. Adjusted linear model for the relationship between the cumulative tobacco exposure and systolic blood pressure in the whole populationa. Web Table 5. Linear and non-linear models for the relationship between the duration of smoking and diastolic blood pressure in the whole population. [file 12889_2020_9975_MOESM1_ESM.docx]

**Relationship between the duration of smoking and blood pressure in Han and ethnic minority populations: a cross-sectional study in China**


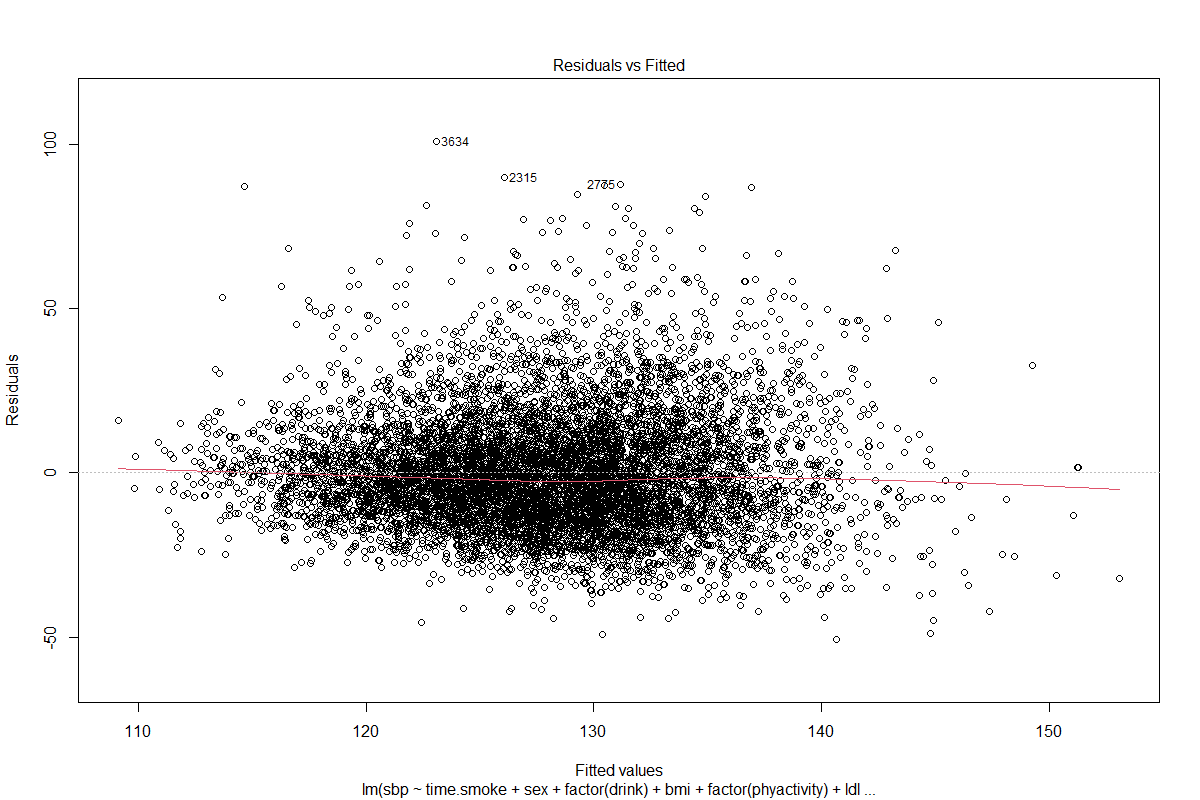


Web Figure 1. Residual plot for checking the assumptions in the linear model in the whole population.

Web Table 1. Questionnaire used in the field survey.

| **Variable name** | **Values or explanations** |
| --- | --- |
| Identification number | Each participant was allocated a unique identification number. |
| Sex | Male or female. |
| Ethnicity | The ethnic characteristics were recorded based on the resident registration in China. |
| Birthday | Date of birth. |
| Field of survey | Name of the community, village, or town where the field survey was completed. |
| Birthplace | The place of birth on the resident registration. |
| Current residency | Current address for home. |
| Educational level | The highest educational level of the participant. |
| Occupation | The primary occupation of the participant. |
| Monthly income | The average monthly income in the past year. |
| Annual income | The annual income in the past year. |
| Household annual income | The annual family income in the past year. |
| Household size | Number of persons living in a household. |
| Hypertension | Yes or no. |
| Highest systolic blood pressure | The highest systolic blood pressure in the past. |
| Highest diastolic blood pressure | The highest diastolic blood pressure in the past. |
| Date of diagnosis of hypertension | Date of diagnosis of hypertension. |
| Healthcare institute, diagnosis of hypertension | The level of healthcare institute where the diagnosis of hypertension was made. |
| Anti-hypertensive drugs | Anti-hypertensive drug therapies. |
| Diabetes | Yes or no. |
| Highest glucose level | The highest glucose in the past. |
| Date of diagnosis of diabetes | Date of diagnosis of diabetes. |
| Healthcare institute, diagnosis of diabetes | The level of healthcare institute where the diagnosis of diabetes was made. |
| Diet | Whether the participant used diet control for diabetes. |
| Exercise for diabetes | Whether the participant had regular exercise for diabetes. |
| Anti-diabetic drugs | Anti-diabetic drug therapies. |
| Insulin | Whether insulin was used to treat diabetes. |
| Traditional Chinese medicine for diabetes | Whether traditional Chinese medicine was used to treat diabetes. |
| No therapy for diabetes | If there was no therapy for diabetes. |
| Anti-diabetic drugs use today | Whether the participant used anti-diabetic drugs on the day of field survey. |
| Smoking | Current, former, or non-smoker. |
| Starting age of smoking | Starting age of smoking. |
| Cigarette consumption per day | Cigarette consumption per day. |
| Tobacco leaf consumption per day | Tobacco leaf consumption per day; the type of tobacco leaf was also recorded. |
| Consumption of other tobacco products | Consumption of other tobacco products (if any). |
| Age at smoking cessation | Age at smoking cessation. |
| Alcohol drinking | Current, former, or non-drinker. |
| Starting age of drinking | Starting age of drinking. |
| Age at drinking cessation | Age at drinking cessation. |
| Sprits consumption per week | The frequency of sprits drinking per week. |
| Sprits consumption per month | The frequency of sprits drinking per month. |
| Sprits consumption per year | The frequency of sprits drinking per year. |
| Average sprits consumption | Average sprits consumption per drink. |
| Wine consumption per week | The frequency of wine drinking per week. |
| Wine consumption per month | The frequency of wine drinking per month. |
| Wine consumption per year | The frequency of wine drinking per year. |
| Average wine consumption | Average wine consumption per drink. |
| Beer consumption per week | The frequency of beer drinking per week. |
| Beer consumption per month | The frequency of beer drinking per month. |
| Beer consumption per year | The frequency of beer drinking per year. |
| Average beer consumption | Average beer consumption per drink. |
| Other alcoholic beverages consumption per week | The frequency of other alcoholic beverages drinking per week. |
| Other alcoholic beverages consumption per month | The frequency of other alcoholic beverages drinking per month. |
| Other alcoholic beverages consumption per year | The frequency of other alcoholic beverages drinking per year. |
| Average consumption of other alcoholic beverages | Average consumption of other alcoholic beverages per drink. |
| Detailed other alcoholic beverages | Name of other alcoholic beverages. |
| Physical activity | Light, moderate, or heavy. |
| Exercise | Frequency of physical exercise. |
| Height | Body height in cm. |
| Weight | Body weight in kg. |
| Grip strength, left | Grip strength of left hand. |
| Grip strength, right | Grip strength of right hand. |
| Reason for using left hand | Reason for using left hand during the measure of grip strength (injuries on right hand or left-handedness). |
| Fasting plasma glucose | Fasting plasma glucose. |
| Total cholesterol | Total cholesterol. |
| Triglyceride | Triglyceride. |
| High-density lipoprotein cholesterol | High-density lipoprotein cholesterol. |
| Low-density lipoprotein cholesterol | Low-density lipoprotein cholesterol. |

Web Table 2. Adjusted models for the duration of smoking and systolic blood pressure in the whole population.

| **Model** | **Estimate** | **95% CI** | ***P*** |
| --- | --- | --- | --- |
| **Spline model** |  |  |  |
| Model 6 |  |  |  |
| Duration of smoking (linear) | 0.048 | -0.022, 0.117 | 0.178 |
| Duration of smoking (non-linear) | 0.345 | 0.267, 0.424 | < 0.001 |
| Sex (male vs. female) | 2.434 | 0.588, 4.279 | 0.010 |
| Quit vs. never drink | 0.682 | -0.615, 1.980 | 0.303 |
| Current vs. never drink | 3.200 | 2.333, 4.067 | < 0.001 |
| BMI | 0.668 | 0.572, 0.764 | < 0.001 |
| Moderate vs. light physical activity | -2.733 | -3.731, -1.734 | < 0.001 |
| Heavy vs. light physical activity | 0.160 | -0.701, 1.021 | 0.716 |
| LDL-C | 1.248 | 0.829, 1.667 | < 0.001 |
| FPG | 1.230 | 0.981, 1.478 | < 0.001 |
| **Segmented model** |  |  |  |
| Model 7^a^ |  |  |  |
| Threshold of duration of smoking | 23.999 | 20.971, 27.028 | -- |

^a^ Adjusted for sex, alcohol drinking, BMI, physical activity, LDL-C, and FPG.

Abbreviations: CI, confidence interval; BMI, body mass index; LDL-C, low-density lipoprotein cholesterol; FPG, fasting plasma glucose.

Web Table 3. Linear regression models for the duration of smoking and systolic blood pressure by quantiles of age.

| **Model** | **Estimate** | **95% CI** | ***P*** |
| --- | --- | --- | --- |
| **Age≤40** |  |  |  |
| Duration of smoking | 0.095 | 0.016, 0.174 | 0.018 |
| Sex (male vs. female) | 7.813 | 4.543, 11.082 | < 0.001 |
| Quit vs. never drink | -0.852 | -3.307, 1.604 | 0.496 |
| Current vs. never drink | 2.341 | 1.111, 3.571 | < 0.001 |
| BMI | 0.760 | 0.631, 0.889 | < 0.001 |
| Moderate vs. light physical activity | -0.753 | -1.945, 0.440 | 0.216 |
| Heavy vs. light physical activity | 0.076 | -1.256, 1.408 | 0.911 |
| LDL-C | 1.301 | 0.710, 1.893 | < 0.001 |
| FPG | 0.446 | -0.052, 0.944 | 0.079 |
| **40<Age≤50** |  |  |  |
| Duration of smoking | -0.009 | -0.104, 0.086 | 0.855 |
| Sex (male vs. female) | 3.463 | -0.064, 6.990 | 0.054 |
| Quit vs. never drink | 0.142 | -2.575, 2.859 | 0.918 |
| Current vs. never drink | 4.020 | 2.395, 5.644 | < 0.001 |
| BMI | 0.724 | 0.551, 0.898 | < 0.001 |
| Moderate vs. light physical activity | -2.638 | -4.383, -0.893 | 0.003 |
| Heavy vs. light physical activity | -1.545 | -3.072, -0.018 | 0.047 |
| LDL-C | 0.702 | -0.043, 1.448 | 0.065 |
| FPG | 1.480 | 0.981, 1.978 | < 0.001 |
| **50<Age≤60** |  |  |  |
| Duration of smoking | 0.012 | -0.070, 0.094 | 0.779 |
| Sex (male vs. female) | 6.991 | 3.369, 10.613 | < 0.001 |
| Quit vs. never drink | 0.449 | -2.121, 3.019 | 0.732 |
| Current vs. never drink | 4.282 | 2.472, 6.092 | < 0.001 |
| BMI | 0.768 | 0.559, 0.978 | < 0.001 |
| Moderate vs. light physical activity | -1.651 | -3.953, 0.652 | 0.160 |
| Heavy vs. light physical activity | 1.043 | -0.658, 2.743 | 0.229 |
| LDL-C | 1.375 | 0.509, 2.241 | 0.002 |
| FPG | 1.139 | 0.719, 1.559 | < 0.001 |
| **Age>60** |  |  |  |
| Duration of smoking | 0.101 | 0.024, 0.178 | 0.010 |
| Sex (male vs. female) | -1.130 | -5.201, 2.940 | 0.586 |
| Quit vs. never drink | -0.170 | -2.797, 2.457 | 0.899 |
| Current vs. never drink | 5.282 | 3.164, 7.400 | < 0.001 |
| BMI | 0.480 | 0.231, 0.729 | < 0.001 |
| Moderate vs. light physical activity | -3.655 | -6.884, -0.425 | 0.027 |
| Heavy vs. light physical activity | 2.579 | 0.393, 4.766 | 0.021 |
| LDL-C | 1.414 | 0.328, 2.500 | 0.011 |
| FPG | 0.650 | 0.065, 1.236 | 0.030 |

Abbreviations: CI, confidence interval; BMI, body mass index; LDL-C, low-density lipoprotein cholesterol; FPG, fasting plasma glucose.

Web Table 4. Adjusted linear model for cumulative tobacco exposure and systolic blood pressure in the whole population ^a^.

| **Variable** | **Estimate** | **95% CI** | ***P*** |
| --- | --- | --- | --- |
| Cumulative dose of tobacco exposure (each 1,000 more cigarettes (50 packs) use per year) | 0.014 | 0.012, 0.017 | < 0.001 |
| Sex (male vs. female) | 1.988 | 0.066, 3.910 | 0.043 |
| Quit vs. never drink | 1.175 | -0.161, 2.511 | 0.085 |
| Current vs. never drink | 2.520 | 1.626, 3.414 | < 0.001 |
| BMI | 0.576 | 0.477, 0.675 | < 0.001 |
| Moderate vs. light physical activity | -3.741 | -4.762, -2.720 | < 0.001 |
| Heavy vs. light physical activity | -0.083 | -0.800, 0.965 | 0.855 |
| LDL-C | 1.366 | 0.935, 1.797 | < 0.001 |
| FPG | 1.421 | 1.168, 1.674 | < 0.001 |

^a^ The sensitivity analysis excluded 249 participants due to the missing data on the dose of tobacco exposure.

Abbreviations: CI, confidence interval; BMI, body mass index; LDL-C, low-density lipoprotein cholesterol; FPG, fasting plasma glucose.

Web Table 5. Models for the duration of smoking and diastolic blood pressure in the whole population.

| **Model** | **Estimate** | **95% CI** | ***P*** |
| --- | --- | --- | --- |
| **Linear model** |  |  |  |
| Model 1 |  |  |  |
| Duration of smoking | 0.086 | 0.067, 0.105 | < 0.001 |
| Model 2 |  |  |  |
| Duration of smoking | 0.105 | 0.086, 0.124 | < 0.001 |
| Sex (male vs. female) | 2.279 | 1.078, 3.480 | < 0.001 |
| Quit vs. never drink | -0.031 | -0.877, 0.813 | 0.941 |
| Current vs. never drink | 2.721 | 2.156, 3.286 | < 0.001 |
| BMI | 0.560 | 0.498, 0.622 | < 0.001 |
| Moderate vs. light physical activity | -1.633 | -2.283, -0.983 | < 0.001 |
| Heavy vs. light physical activity | -1.639 | -2.194, -1.083 | < 0.001 |
| Model 3 |  |  |  |
| Duration of smoking | 0.088 | 0.069, 0.107 | < 0.001 |
| Sex (male vs. female) | 2.596 | 1.400, 3.791 | < 0.001 |
| Quit vs. never drink | -0.308 | -1.149, 0.534 | 0.474 |
| Current vs. never drink | 2.692 | 2.131, 3.254 | < 0.001 |
| BMI | 0.517 | 0.454, 0.579 | < 0.001 |
| Moderate vs. light physical activity | -1.488 | -2.134, -0.841 | < 0.001 |
| Heavy vs. light physical activity | -1.318 | -1.873, -0.763 | < 0.001 |
| LDL-C | 0.978 | 0.706, 1.249 | < 0.001 |
| FPG | 0.657 | 0.496, 0.817 | < 0.001 |
| **Spline model** |  |  |  |
| Model 4^a^ |  |  |  |
| Duration of smoking (linear) | 0.223 | 0.178, 0.268 | < 0.001 |
| Duration of smoking (non-linear) | -0.168 | -0.219, -0.117 | < 0.001 |
| **Segmented model** |  |  |  |
| Model 5 ^a^ |  |  |  |
| Threshold of duration of smoking | 28.000 | 24.144, 31.856 | -- |
| Duration of smoking (shorter than 28.000 years) | 0.184 | 0.144, 0.225 | < 0.001 |
| Duration of smoking (longer than 28.000 years) | -0.030 | -0.081, 0.020 | -- |

^a^ Fully adjusted model in which sex, alcohol drinking, BMI, physical activity, LDL-C, and FPG were controlled.

Abbreviations: CI, confidence interval; BMI, body mass index; LDL-C, low-density lipoprotein cholesterol; FPG, fasting plasma glucose.
